# Innovations for scaling up family medicine training within the Primary Care and Family Medicine network

**DOI:** 10.4102/phcfm.v18i1.5427

**Published:** 2026-06-30

**Authors:** Innocent K. Besigye, Robert Mash

**Affiliations:** 1 Department of Family Medicine, School of Medicine, Makerere University, Kampala, Uganda; 2 Division of Family Medicine and Primary Care, Faculty of Health Sciences, Stellenbosch University, Cape Town, South Africa

## Context

Sub-Saharan Africa (SSA) continues to struggle to scale up family medicine training, with some countries not yet embracing family medicine at all. Even in countries where training exists, not every medical school has postgraduate family medicine training programmes. At the few medical schools with family medicine departments and training, recruitment of trainees is sub-optimal, and training opportunities may be scarce. This undermines the vision of achieving a sufficient number of family physicians to strengthen primary care and primary hospitals across the region of over 1.6 billion people.^1^ The academic departments of family medicine need to learn from each other on how to scale up postgraduate training to maximise their training potential, and for family physicians to contribute to strengthening district-level health services.

The Primary Care and Family Medicine (PRIMAFAMED) network annually brings together family medicine educators from more than 40 academic departments of family medicine from across more than 25 countries in SSA. During these annual workshops, educational and research capacity-building is conducted. The workshops provide opportunities to share experiences and learn together through invited poster presentations. The 2-day workshop took place on 24–25 June 2025 at Lusaka in Zambia.

## Poster presentations

Each institution was requested to prepare a poster presentation describing any innovative strategies and lessons learned in scaling up with postgraduate family medicine training. All the posters submitted and presented are freely available online.^2^ The authors performed a descriptive analysis of the posters, highlighting the focus area, problems addressed, innovations instituted, and the outcomes of the innovations. The results are summarised in Table 1.

**TABLE 1:** Poster descriptive analysis results.

University	Focus area	Problem addressed	Innovation	Outcome
Sefako Makgatho Health Sciences University (South Africa)	Teaching and learning	Inadequate learner-centred research education strategies	Compulsory online research methodology training and assessment for first-year registrars Compulsory rotation of first-year registrars in a nearby research centre	All first-year registrars developed their research proposals within 3 months of registration with the university
Aga Khan University, Nairobi (Kenya)	Student support, personal growth and wellbeing	Feelings of incompetence, isolation and burn out as well as well as mistreatment, stigma and reluctancy to use mental health services	Peer and mentor support and team-building exercises Improved access to mental health services and opportunities for confidential psychological assessments	No resident attrition in the first-year, better academic progression and all finalists graduating
Ghana College of Physicians and Surgeons (Ghana)	Development of family medicine training programmes	Potential trainees failing to leave their workplaces or private practices to undergo training	A modular family medicine programme was designed. This allowed trainees to continue with their work as they trained	Increased enrolment into the training
University of Zambia (Zambia)	Advocacy for family medicine	Limited understanding of family medicine’s role by the different stakeholders	Extensive advocacy for buy-in of stakeholders	Inclusion of family medicine in the national health policy, with commitment to posts and career progression
Makerere University (Uganda)	Development of family medicine training programmes	Lack of family medicine in undergraduate curriculum	Introduction of family medicine in undergraduate curriculum	Increased number of applicants for family medicine postgraduate training
Kamuzu University (Malawi)	Advocacy for family medicine	Limited knowledge and awareness of family medicine	In-person and online advocacy talk about family medicine at district and central hospitals	An increased number of trainees recruited
University of Cape Town (South Africa)	Teaching and learning	Contact sessions had variable quality and alignment with learning needs	Co-creation of teaching, learning, and assessment by faculty and registrars	Empowered registrars as adult self-directed learners
Aga Khan University (Tanzania)	Development of family medicine training programmes	No family medicine in public universities and government health services	Training programme is fully sponsored in private sector with shift from hospital to primary care exposure	Graduates registered as family physicians but only work within the private sector
Moi University (Kenya)	Advocacy for family medicine	Low numbers of trainees and employer resistance to releasing medical officers for postgraduate training	Engagement of government and the private sector, and modification of the programme by opening two new sites	Increased number of family medicine postgraduate trainees
Stellenbosch University (South Africa)	Student support, personal growth, and well-being	Personal and professional growth needed for leadership	Group coaching of registrars focusing on personal and professional growth	Graduates with personal growth and leadership skills
University of the Gambia (The Gambia)	Advocacy for family medicine	New programme largely unknown in the country	Engagement of stakeholders, including policymakers, academics, and undergraduate students	Improved awareness of the specialty and an increased number of trainees recruited
Protestant University of Congo (DRC)	Advocacy for family medicine	Lack of family medicine recognition in the country	Defining the family medicine career path and supporting other universities to start family medicine training	Recognition of family medicine by the National Council of Medicine
University of Limpopo (South Africa)	Development of family medicine training programmes	Weak supportive environment for learning	Structured programme with regular group contact and regular feedback on progress in a nurturing environment	Increased number of trainees retained in the programme
University of Namibia (Namibia)	Development of family medicine training programmes	Unclear implementation of the new family medicine programme	Early undergraduate exposure to family medicine and introduction of diploma in family medicine	Improved interest in family medicine and its footprint in Namibia
Amoud University (Somaliland)	Teaching and learning	Poor chronic care for non-communicable diseases	Embedded quality improvement in clinical training	Enhanced clinical skills for non-communicable disease care among the trainees
Eduardo Mondlane University (Mozambique)	Development of family medicine training programmes	Urban family medicine training, but no rural training opportunities	Partnership with a non-government organisation to extend family medicine training into rural areas	Competent fit-for-purpose family physicians
Maseno University (Kenya)	Advocacy for family medicine	Family medicine is a new specialty and largely unknown	Engagement of students, doctors and partners	Increased number of trainees recruited and graduating
Federal Medical Centre (Nigeria)	Student support, personal growth, and well-being	Slowly progressing trainees	Supportive mentoring to struggling trainees	Decreased dropout of trainees
University of Botswana (Botswana)	Development of family medicine training programmes	Low family medicine faculty numbers	Faculty size expanded through the appointment of adjunct lecturers	An increased number of trainees are enrolled and graduating
South Sudan	Advocacy for family medicine	No family medicine in the country	Local champions spearheading family medicine advocacy through stakeholder engagement and collaborative partnerships	Country vision for family medicine
University of Pretoria (South Africa)	Development of family medicine training programmes	Inappropriate training environment for relevant competences	Community-based postgraduate family medicine training focusing on graduate expertise in generalist comprehensive care	Fit-for-purpose graduates

DRC, Democratic Republic of the Congo.

## Discussion

Innovations and activities to strengthen and scale up family medicine training fell into four categories related to the stage of family medicine development in the country: advocacy for family medicine, development of family medicine training programmes, teaching and learning, student support, personal growth and well-being.

Several countries were still advocating for the establishment of family medicine training (e.g. South Sudan, The Gambia). Countries engaged with a variety of stakeholders and with different intentions. For example, advocacy with government to include family medicine in policy and create posts for family physicians (e.g. Zambia), with other disciplines and doctors to enhance understanding and increase interest in the discipline (e.g. Kenya), with private and public sector decision makers to release doctors to train in family medicine (e.g. Kenya), or with higher education institutions to start postgraduate training (e.g. Democratic Republic of the Congo).

In countries that had decided to adopt training in family medicine, there was a focus on consolidating, improving, and extending the training programmes. For example, extending family medicine to the undergraduate programme (e.g. Uganda), developing a diploma in family medicine (e.g. Namibia), changing the structure of the programme to better align with the needs of employers and students (e.g. Ghana), or creating a better learning environment (e.g. South Africa). Where the public sector was not interested, one programme was fully developed in the private sector (e.g. Tanzania). Others formed new partnerships in the non-government sector to expand training in rural areas (e.g. Mozambique), while others increased the number of faculty and trainers (e.g. Botswana).

Established programmes often had a focus on improving teaching and learning. For example, improving the teaching of research through new modules and practical exposure (e.g. South Africa); embracing adult learning by co-creating contact-session teaching and self-directed learning (e.g. South Africa); and using service learning by engaging quality improvement cycles (e.g. Somaliland). Several programmes also focused on student support, personal growth, and wellbeing to improve retention, performance, and appropriate leadership development. This was done through mentoring (e.g. Nigeria), group coaching (e.g. South Africa) and better access to mental health counselling and services (e.g. Kenya).

As the region continues to develop and scale up family medicine, institutions and countries should continue learning from each other. Therefore, such workshops remain a valuable opportunity to share experiences and lessons. The innovations described in the poster presentations are not new, as they have been tried before. Since the 1990s, SSA has continued to develop and scale up family medicine in innovative ways. Innovative approaches that have been used include: shortened training time at Gezira University in Sudan,^3^ use of non-family physician champions in Uganda and Ethiopia,^4,5^ different advocacy approaches,^6^ modification of learning environment to produce fit-for-purpose graduates, and the use of distance education programmes.^7^ All these innovative efforts have contributed to the progressive increase in the number of family physicians within the region. Partnerships using North-South and South-South collaborations have contributed significantly to the innovative scale up of family medicine in SSA.^8^

## Identified gaps in the innovations

Faculty development does not feature much in the innovations. Family medicine faculty are limited in number and often quite junior within the SSA region, which becomes a constraint on scaling up training. The Family Medicine Leadership Education and Assessment Programme project trained a significant number of clinical trainers but was not sustained.^9^

Very few institutions described innovations in curriculum design and implementation. Any successful academic programme requires a well-thought-through curriculum that is regularly revised to cater for new and emerging disease conditions, technological or educational innovations, and new knowledge in the field. When a country has multiple training programmes there is also a need to agree on national learning outcomes so that all programmes produce similar fit-for-purpose graduates. This limited focus on curricular innovations may be due to some curricula being relatively new, but there is a risk of stagnation and of producing graduates unable to deal with contemporary challenges.

The innovations reported by the different institutions fell short of digital technologies. A few of the institutions reported using online or electronic innovations to scale up postgraduate family medicine training. No institution mentioned the use of artificial intelligence.

## Implications

Advocacy is still a major issue in the development of family medicine training, and further training in advocacy and leadership may be needed. World Organization of Family Doctors (WONCA) Africa has identified this as a need.^10^ The need to develop a pipeline from undergraduate training to internship and then postgraduate training has been well recognised. The value of shorter non-specialist postgraduate training through diplomas for established medical officers and general practitioners has also been previously identified.^11^ The discipline should therefore develop a pipeline and menu of training options when they have sufficient capacity.

Expanding the number of training institutions and sites is a clear issue, and the newly established East, Central, and Southern African College of Family Physicians intends to address this across 11 countries.^12^ They will create additional training opportunities alongside the university-based programmes. Attention to training in both rural and urban settings is a key issue for deploying family physicians and reducing maldistribution within a country.

Innovations should include a special focus on faculty and clinical trainer development. At higher education institutions, faculty need skills in curriculum design, workplace-based and traditional assessment, digital and online teaching technologies, as well as leadership and management capabilities. Artificial intelligence will transform the teaching and learning environment in the future, and while it can enhance learning, it also poses ethical and assessment challenges. As training programmes require research, each programme should develop established researchers who can provide effective supervision, for example, through doctoral training programmes. Clinical trainers need to develop their educational toolbox in workplace-based training and assessment alongside their clinical competencies. The young doctors’ movement in Africa has developed an e-mentorship programme (The Afriwon Research Collaborative) to support research proposal writing.^13^ Stellenbosch University also offers a non-clinical Master’s degree to enable faculty development, but the numbers are small. There is a need to re-establish training of clinical trainer programmes such as the national 5-day course in South Africa.

Regional and national stakeholder engagement is needed to ensure that training programmes remain dynamic and aligned with the needs of health systems and the communities served. Training programmes should also endeavour to engage with the communities where they train and where family physicians are deployed to increase awareness among the public of family medicine and to obtain meaningful feedback on how to improve the training of family physicians. This is still aspirational for most programmes.

## Conclusion

Most institutions within the PRIMAFAMED network are innovatively scaling up family medicine postgraduate training. The innovations have increased the number of trainees and graduates as well as awareness of the role of family medicine for health systems in SSA. However, there is a need to continue to support advocacy and leadership, expand the training pipeline and menu of training options, develop faculty and clinical trainers, embrace digital technology and artificial intelligence, and support students in their personal and professional growth.
